# Supplementary material for: Independent Innexin Radiation Shaped Signaling in Ctenophores
Source: Mol Biol Evol. 2023 Feb 6;40(2):msad025. doi: 10.1093/molbev/msad025 (PMC9949713; doi:10.1093/molbev/msad025)
Supplement: msad025_Supplementary_Data [file msad025_supplementary_data.docx]

**
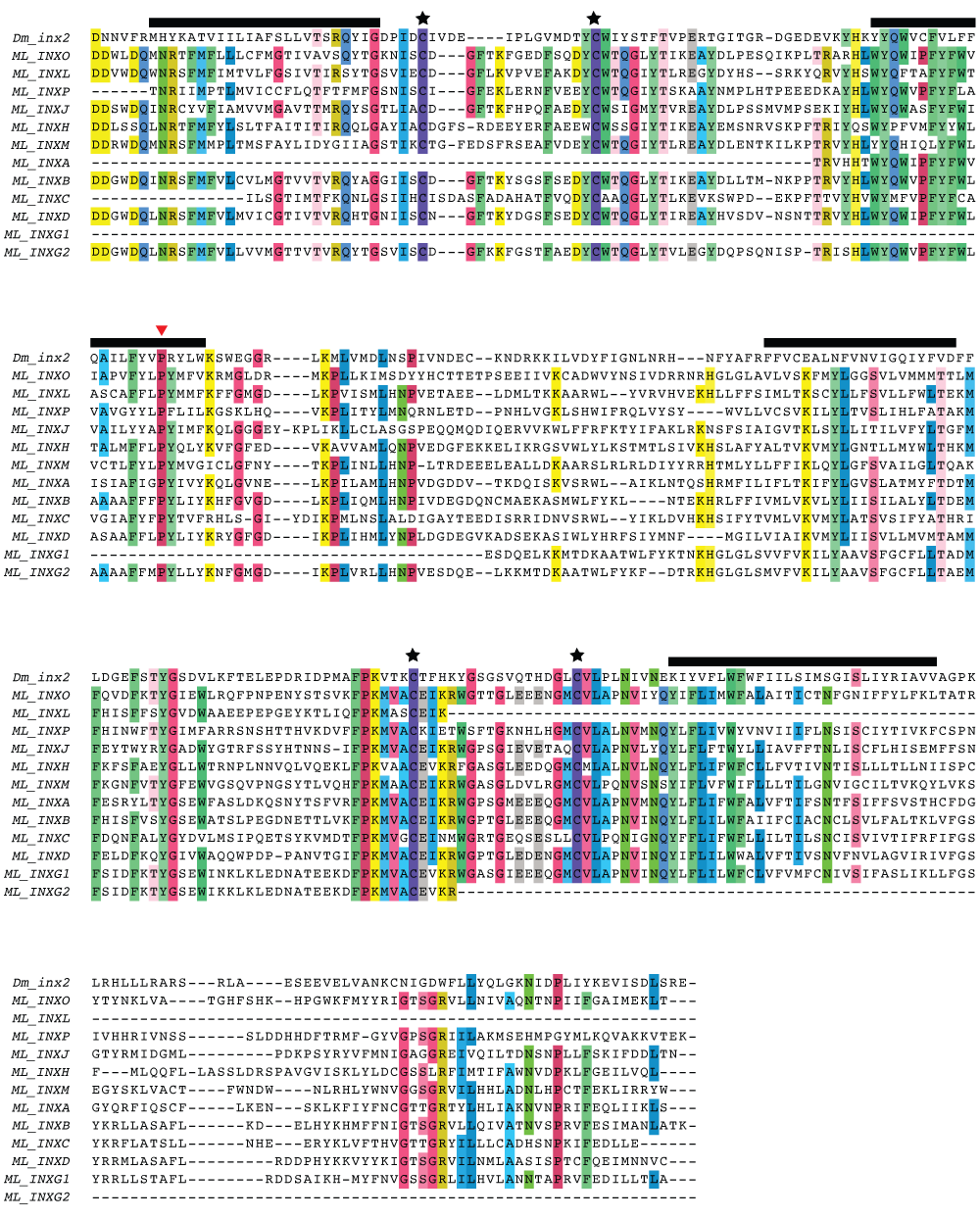
**

**Supplementary Figure 1. Alignment of *Drosophila* *melanogaster* Inx-2 and all of the *Mnemiopsis leidyi* innexins reveals conserved residues.** Conserved features of *Drosophila melanagoster* Inx2 (Dm_Inx2) are indicated above the alignment (after Tazuke et al. 2002). Extracellular cysteine residues are indicated by stars and conserved proline in the second transmembrane domain are indicated by a red triangle. The N-terminal and C-terminal ends of these sequences are not shown and were not used in the phylogenetic analyses. A background color is used for columns where 50% or more of residues are the same (default colors used in Unipro UGENE).

**
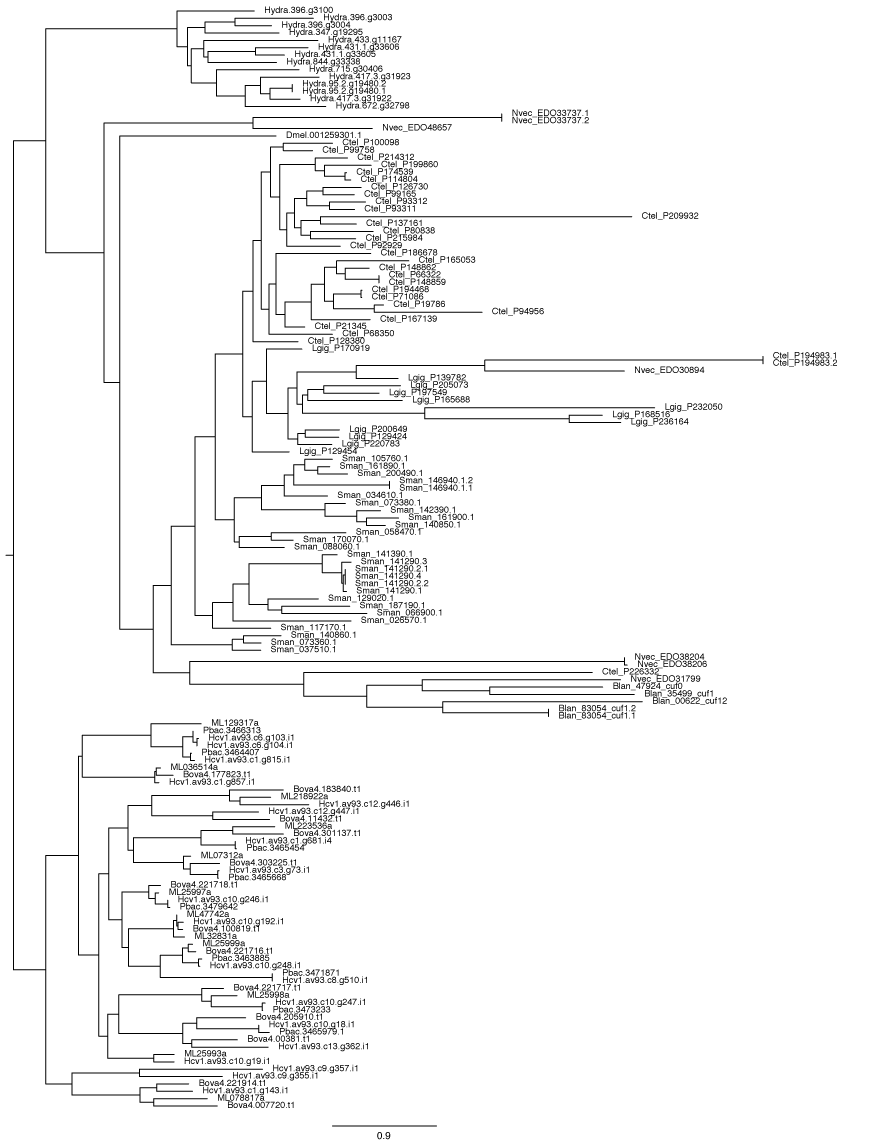
**

**Supplementary Figure 2. Detailed maximum-likelihood phylogeny of innexins.** This tree is summarized in Figure 2 of the main manuscript. Bootstrap values below 70 are regarded as suspect and below 50 are regarded as unreliable. It is included to show details of collapsed clades. A newick version of this tree (6taxa_plus_hcal_genome_plus_bilat.pruned.tre) is available in the Github repository associated with this study.

**
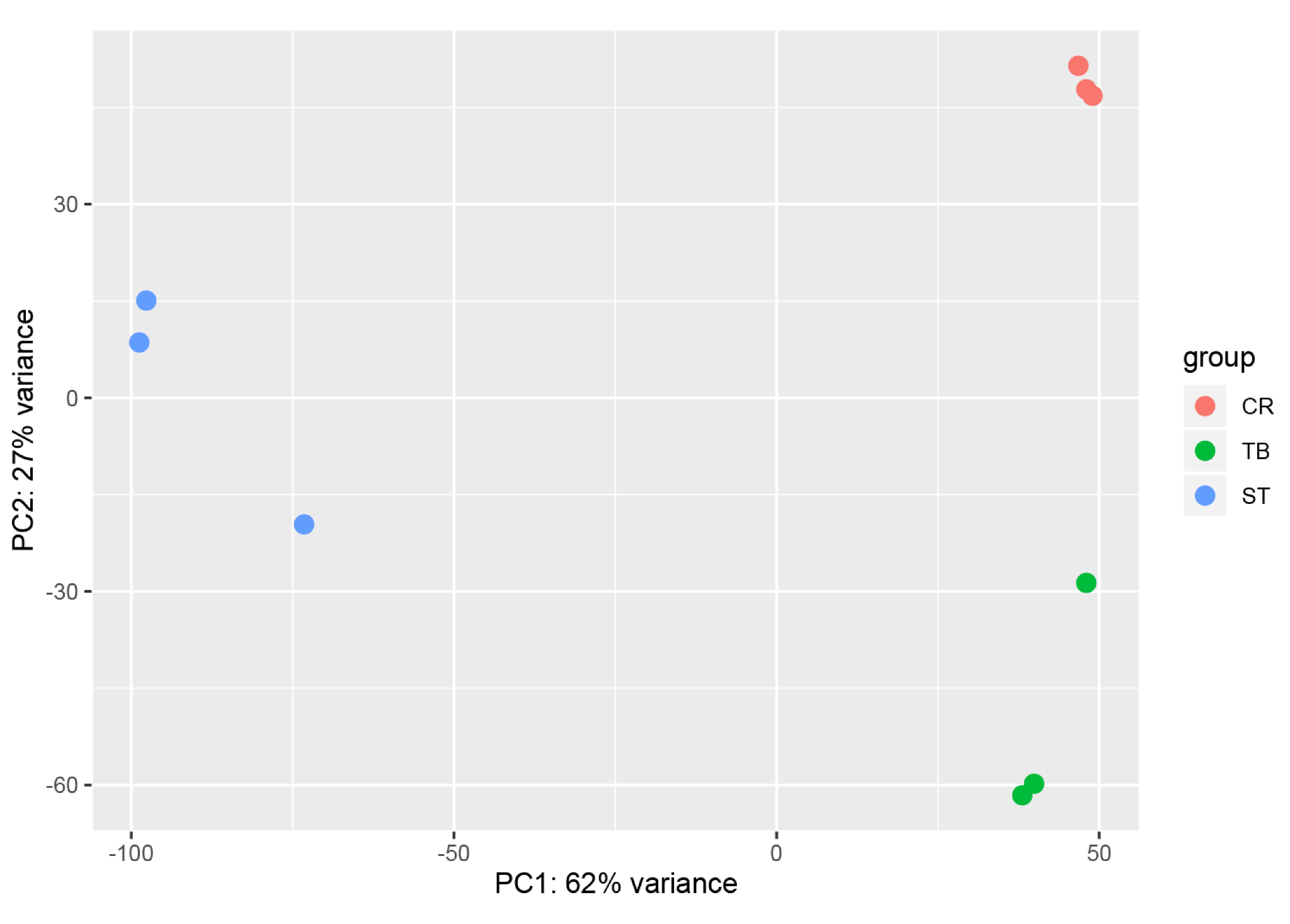
**

**Supplementary Figure 3. Principal components analysis of tissue RNA-Seq data.** PCA was performed using plotPCA function in DESeq2 on genes with counts in at least 3 libraries. CR=comb row, TB=tentacle bulb, ST=statocyst.

**
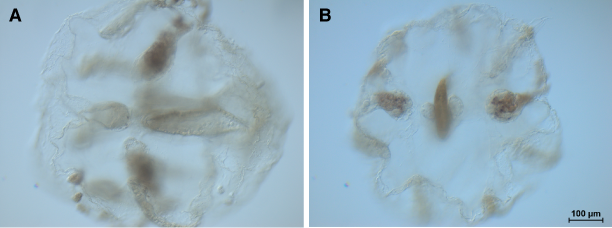
**

**Supplementary Figure 4. No probe controls for whole mount in situ hybridization.** Slight background staining can be seen in pharynx, tentacle, and tentacle bulb tissue. (A) lateral view, (B) aboral view.


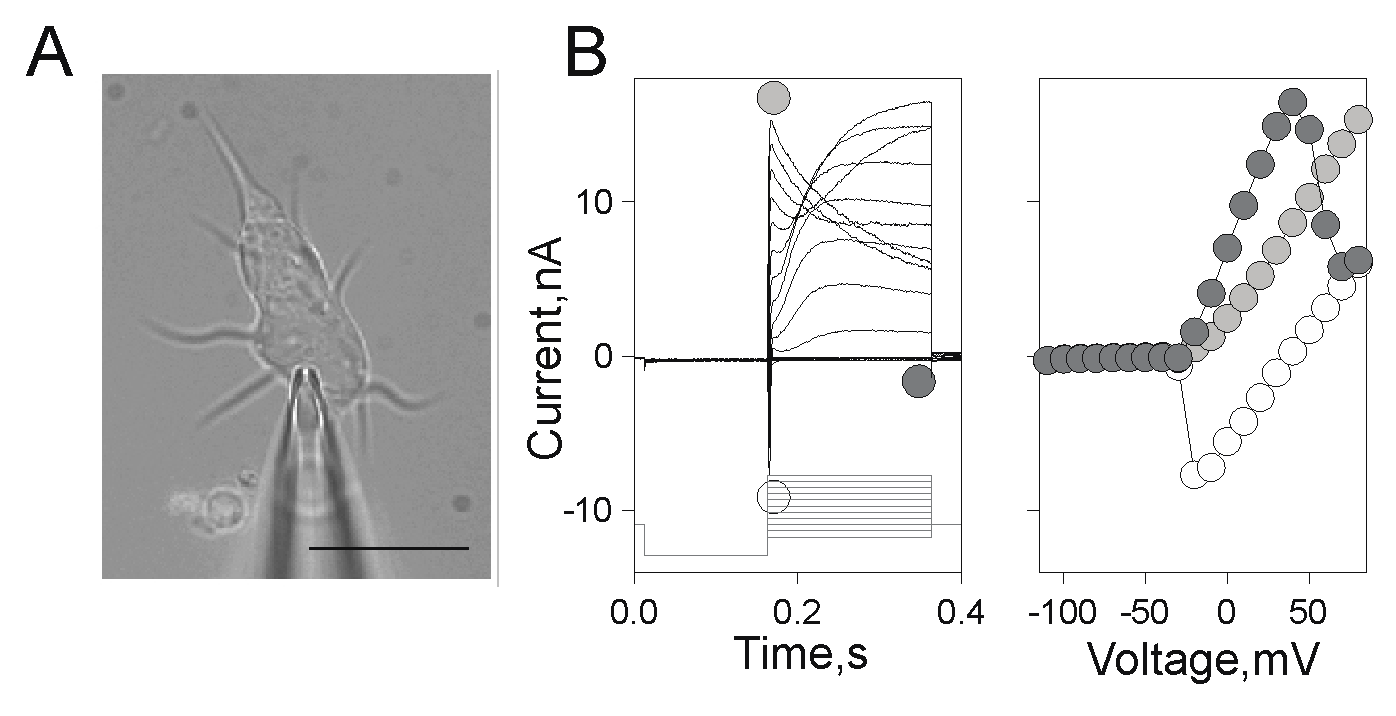


**Supplemental Figure 5. General characteristics of the ctenophore *Mnemiopsis leidyi* muscle cell conductances**. (A) Muscle cell/muscle cell fragment in the ctenophore primary cell culture. (B) Typical set of voltage-dependent currents recorded from isolated muscle cells using whole-cell voltage-clamp recording: Inward currents characterized by fast activation/inactivation kinetics (white circle/s) represent activity of voltage-gated sodium channels. Fast activating outward currents (A type currents, grey circle/s) are presumably mediated by the activity of delayed rectifier potassium channels. And, relatively slowly activating outward currents (dark grey circle/s) reflect the activity of voltage-gated, calcium sensitive potassium channels. (B, right panel) Respective current-voltage characteristics. Current scales in B are the same. Scale bar 20µm. A and B represent different cells.

**
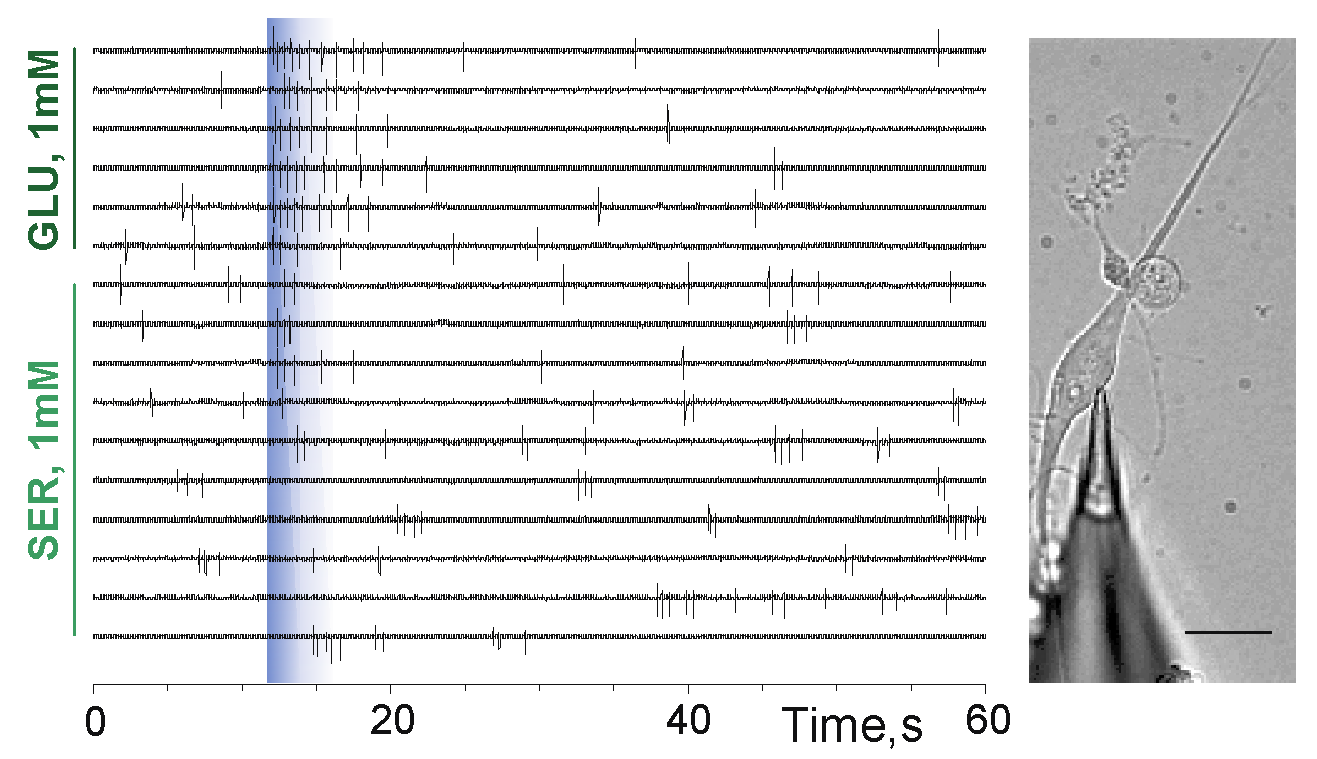
Supplemental Figure 6.** **Ligand evoked activity of isolated ctenophore *Mnemiopsis leidyi* muscle cells.** Typical responses of ctenophore muscle cells to 500ms pulses of a transmitter application (blue faded bar) were recorded using loose-patch clamp configuration. These cells are sensitive to glutamate and capable of generating action potentials in a consistent manner. Glutamate repetitively increases ongoing rate of discharge. Multi-channel rapid solution changer (RSC-160, Bio-Logic) under software control (Clampex 9/10, Molecular Devices) was used for neurotransmitter/s application. Simultaneous video recording (Supplemental Video 1) shows evoked contractile activity of the cell. Scale bar 20µm. GLU=Glutamate; SER=Serine.

**Supplementary Table 1. Peer reviewed publications providing phylogenomic evidence supporting ctenophores as the sister group to the rest of animals**

| Dunn CW, Hejnol A, Matus DQ, Pang K, Browne WE, Smith SA, Seaver E, Rouse GW, Obst M, Edgecombe GD, Sørensen MV, Haddock SH, Schmidt-Rhaesa A, Okusu A, Kristensen RM, Wheeler WC, Martindale MQ, Giribet G. Broad phylogenomic sampling improves resolution of the animal tree of life. Nature. 2008 Apr 10;452(7188):745-9. doi: 10.1038/nature06614 |
| --- |
| Hejnol A, Obst M, Stamatakis A, Ott M, Rouse GW, Edgecombe GD, Martinez P, Baguñà J, Bailly X, Jondelius U, Wiens M, Müller WE, Seaver E, Wheeler WC, Martindale MQ, Giribet G, Dunn CW. Assessing the root of bilaterian animals with scalable phylogenomic methods. Proc Biol Sci. 2009 Dec 22;276(1677):4261-70. doi: 10.1098/rspb.2009.0896. |
| Ryan JF, Pang K, Schnitzler CE, Nguyen AD, Moreland RT, Simmons DK, Koch BJ, Francis WR, Havlak P; NISC Comparative Sequencing Program, Smith SA, Putnam NH, Haddock SH, Dunn CW, Wolfsberg TG, Mullikin JC, Martindale MQ, Baxevanis AD. The genome of the ctenophore Mnemiopsis leidyi and its implications for cell type evolution. Science. 2013 Dec 13;342(6164):1242592. doi: 10.1126/science.1242592 |
| Moroz LL, Kocot KM, Citarella MR, Dosung S, Norekian TP, Povolotskaya IS, Grigorenko AP, Dailey C, Berezikov E, Buckley KM, Ptitsyn A, Reshetov D, Mukherjee K, Moroz TP, Bobkova Y, Yu F, Kapitonov VV, Jurka J, Bobkov YV, Swore JJ, Girardo DO, Fodor A, Gusev F, Sanford R, Bruders R, Kittler E, Mills CE, Rast JP, Derelle R, Solovyev VV, Kondrashov FA, Swalla BJ, Sweedler JV, Rogaev EI, Halanych KM, Kohn AB. The ctenophore genome and the evolutionary origins of neural systems. Nature. 2014 Jun 5;510(7503):109-14. doi: 10.1038/nature13400 |
| Chang ES, Neuhof M, Rubinstein ND, Diamant A, Philippe H, Huchon D, Cartwright P. Genomic insights into the evolutionary origin of Myxozoa within Cnidaria. Proc Natl Acad Sci U S A. 2015 Dec 1;112(48):14912-7. doi: 10.1073/pnas.1511468112 |
| Whelan NV, Kocot KM, Moroz LL, Halanych KM. Error, signal, and the placement of Ctenophora sister to all other animals. Proc Natl Acad Sci U S A. 2015 May 5;112(18):5773-8. doi: 10.1073/pnas.1503453112 |
| Torruella G, de Mendoza A, Grau-Bové X, Antó M, Chaplin MA, del Campo J, Eme L, Pérez-Cordón G, Whipps CM, Nichols KM, Paley R, Roger AJ, Sitjà-Bobadilla A, Donachie S, Ruiz-Trillo I. Phylogenomics Reveals Convergent Evolution of Lifestyles in Close Relatives of Animals and Fungi. Curr Biol. 2015 Sep 21;25(18):2404-10. doi: 10.1016/j.cub.2015.07.053 |
| Borowiec ML, Lee EK, Chiu JC, Plachetzki DC. Extracting phylogenetic signal and accounting for bias in whole-genome data sets supports the Ctenophora as sister to remaining Metazoa. BMC Genomics. 2015 Nov 23;16:987. doi: 10.1186/s12864-015-2146-4 |
| Arcila D, Ortí G, Vari R, Armbruster JW, Stiassny MLJ, Ko KD, Sabaj MH, Lundberg J, Revell LJ, Betancur-R R. Genome-wide interrogation advances resolution of recalcitrant groups in the tree of life. Nat Ecol Evol. 2017 Jan 13;1(2):20. doi: 10.1038/s41559-016-0020 |
| Whelan NV, Kocot KM, Moroz TP, Mukherjee K, Williams P, Paulay G, Moroz LL, Halanych KM. Ctenophore relationships and their placement as the sister group to all other animals. Nat Ecol Evol. 2017 Nov;1(11):1737-1746. doi: 10.1038/s41559-017-0331-3 |
| Shen XX, Hittinger CT, Rokas A. Contentious relationships in phylogenomic studies can be driven by a handful of genes. Nat Ecol Evol. 2017 Apr 10;1(5):126. doi: 10.1038/s41559-017-0126 |
| Laumer CE, Fernández R, Lemer S, Combosch D, Kocot KM, Riesgo A, Andrade SCS, Sterrer W, Sørensen MV, Giribet G. Revisiting metazoan phylogeny with genomic sampling of all phyla. Proc Biol Sci. 2019 Jul 10;286(1906):20190831. doi: 10.1098/rspb.2019.0831 |
| Jeon Y, Park SG, Lee N, Weber JA, Kim HS, Hwang SJ, Woo S, Kim HM, Bhak Y, Jeon S, Lee N, Jo Y, Blazyte A, Ryu T, Cho YS, Kim H, Lee JH, Yim HS, Bhak J, Yum S. The Draft Genome of an Octocoral, Dendronephthya gigantea. Genome Biol Evol. 2019 Mar 1;11(3):949-953. doi: 10.1093/gbe/evz043 |
| Kim HM, Weber JA, Lee N, Park SG, Cho YS, Bhak Y, Lee N, Jeon Y, Jeon S, Luria V, Karger A, Kirschner MW, Jo YJ, Woo S, Shin K, Chung O, Ryu JC, Yim HS, Lee JH, Edwards JS, Manica A, Bhak J, Yum S. The genome of the giant Nomura's jellyfish sheds light on the early evolution of active predation. BMC Biol. 2019 Mar 29;17(1):28. doi: 10.1186/s12915-019-0643-7 |
| Erives A, Fritzsch B. A Screen for Gene Paralogies Delineating Evolutionary Branching Order of Early Metazoa. G3 (Bethesda). 2020 Feb 6;10(2):811-826. doi: 10.1534/g3.119.400951 |
| Pandey A, Braun EL. Phylogenetic Analyses of Sites in Different Protein Structural Environments Result in Distinct Placements of the Metazoan Root. Biology (Basel). 2020 Mar 28;9(4):64. doi: 10.3390/biology9040064 |
| Li Y, Shen XX, Evans B, Dunn CW, Rokas A. Rooting the Animal Tree of Life. Mol Biol Evol. 2021 Sep 27;38(10):4322-4333. doi: 10.1093/molbev/msab170 |

**Supplementary Table 2.** Probe sequences used for whole mount in situ hybridization:

| INXA  (GC: 39.45%) | ATGTTATTGG AGATATTAGC GAACTTCAAA GGAGCGACAC CTTTCAAAGA AATAGTTCTA  GATGACAAGT GGGACCAGAT TAACCGATGT TACATGTTCC TGCTGTGTGT GATTTTCGGA  ACTGTCGGAC TGCTACCGGA AGATGCGCCT CCCTGTCTCT CCAGACGATT AGTGTCTGGT  GGAAGAATAG AATGTCCTCC TGCTGACCTG TACTTGGAAC CAACAAGGGT TCATCACACA  TGGTATCAGT GGATACCGTT TTACTTTTGG GTCATATCCA TAGCGTTCAT TGGTCCTTAC  ATAGTCTACA AGCAGCTGGG TGTCAACGAA CTGAAGCCTA TTCTGGCAAT GCTTCATAAC  CCGGTTGATG GTGACGATGT TACAAAGGAT CAAATAAGCA AAGTCTCAAG ATGGTTAGCT  ATCAAGCTGA ACATCTTTAT CCAAGAAAAA TCTACCTATG CCAAGATCAC TCAGAGCCAT  AGGATGTTTA TTCTAATCTT TTTAACTAAA ATATTCTATC TTGGAGTAAG TTTGGCTACA  ATGTATTTTA CTGACACCAT GTTTGAATCC GGCCGCTACC TTACTTACGG CAGCGAATGG  TTCGCATCTC TCGATAAGCA ATCAAACTAC ACAAGTTTTG TGCGAGACAG ACTGTTCCCG  AAAATGGTGG CATGTGAGAT CAAGAGATGG GGTCCTTCAG GTATGGAGGA GGAACAAGGG  ATGTGTGTTC TTGCTCCGAA CGTGATGAAC CAATATCTCT TCCTCATCTT CTGGTTCGCA  CTCGTCTTCA CCATCTTCTC CAACACCTTC TCCATCTTCT TCTCCGTTTC GACCCACTGT  TTTATTGACG GTGGGTACCA GAGGTTTATC CAGAGCTGCT TTCTAAAAGA AAACAGCAAA  CTGAAGTTCA TCTATTTCAA TTGTGGGACT ACTGGCCGGA CTTATCTGCA TCTAATTGCC  AAAAACGTTA ACCCTCGGAT TTTTGAACAG CTCATCATCA AACTTAGTGC AGATTTAGTT  GAGGAGAAAA ATAAGCAACA CTTAAAGGGG TCAAAGGACA TACTAGTTTG A |
| --- | --- |
| INXB  (GC: 45.84%) | ATGGTTATTG ACATCCTCTC CGGTTTTAAG GGGATCACGC CCTTCAAAGG CATCACTTTA  GACGATGGAT GGGATCAAAT CAACAGGAGT TTTATGTTCG TTCTGTGCGT TTTAATGGGA  ACGGTGGTGA CGGTGCGGCA GTACGCGGGA GGAATTATAT CATGTGACGG CTTCACCAAA  TACTCGGGCT CGTTTAGCGA GGATTACTGT TGGACACAGG GTCTTTACAC CATCAAGGAA  GCCTATGATC TCCTCACCAT GAATGTGCCT TATCCAGGTG TCATCCCTGA GGACATGCCC  ACCTGTATCG AGCGGGAACT TATAAATGGA GGCCGAGTGT CCTGCCCCGA CCCTGAAACA  GTCAAACCAC CCACAAGAGT CTACCACTTA TGGTACCAAT GGGTTCCATT CTACTTCTGG  CTCGCAGCTG CCGCCTTCTT CTTCCCTTAT CTCATCTACA AACATTTCGG CGTTGGCGAC  CTCAAACCCC TCATCCAAAT GCTCCACAAC CCTATTGTTG ACGAAGGTGA CCAGAACTGC  ATGGCCGAAA AAGCTAGCAT GTGGCTCTTC TACAAACTGA ACGTTTTCAT GAACGAGAAC  ACAATCTTCG CTATCCTGAC TGAAAAACAC CGACTTTTCT TCATTGTTAT GCTCGTCAAG  GTGCTCTATC TGATCATCAG TATTCTGGCC CTCTACCTTA CGGACGAGAT GTTCCACATC  GGCTCCTTTG TCTCGTACGG GAGCGAGTGG GCGACCTCCC TGCCCGAGGG AGACAACGAG  ACGACTCTCG TTAAAGACAA ACTCTTCCCC AAGATGGTCG CTTGTGAGAT CAAACGATGG  GGACCTACCG GTCTCGAGGA GGAACAGGGC ATGTGTGTGT TGGCTCCCAA CGTTATCAAC  CAATATCTCT TCCTCATCCT CTGGTTCGCC ATTATTTTCT GCATTGCTTG CAACTGTTTG  TCCGTCCTCT TCGCCCTTAC AAAGCTGGTC TTCGTCTTGG GCTCCTACAA GAGGCTCCTA  GCCAGTGCTT TCCTCAAGGA TGAACTCCAC TACAAACACA TGTTCTTCAA CATTGGTACA  AGTGGACGAG TTCTCCTACA AATCGTTGCG ACGAATGTGT CACCGCGGGT CTTCGAGTCC  ATCATGGCTA ACCTTGCGAC CAAGTTGATA GCTGAACGTT TGAAGGGAAA CGGCAAAGGT  AGCGTCTAG |
| INXC  (GC: 41.58%) | ATGTTTTGTA TTTTATCTGG TACAATCATG ACCTTTAAAC AGAATTTAGG ATCAATAATA  CACTGTATAT CGGATGCAAG AGGCGACGAC AGTTCGTTTG CGGATGCTCA TGCGACATTT  GTGCAAGACT ATTGTGCTGC TCAAGGGCTG TACACTTTAA AAGAAGTGTA TGACAAGTCT  TGGCCAGATG AAATTCCTTA CCCAGGTATT CTCCAAATGA AAACAATCGG TTGTTTCCCG  GGGAGACAGT TCAAAAACGG AACCCCCATC CAGTGCCCGG ACGAGAAAGA TCTGAAACCC  TTCACAACGG TCTATCATGT CTGGTACATG TTCGTACCGT TCTACTTCTG CGCTGTTGGC  ATCGCTTTTT ACTTCCCCTA CACGGTTTTC AGACACCTCA GCGGCATCTA CGACATCAAG  CCTATGTTGA ACAGCCTTGC CCTCGACATT GGGGCCTACA CGGAGGAGGA CATAAGTCGA  CGTATAGACA ATGTCTCGAG GTGGTTGTAC ATCAAGTTGG ATCCCTACAT GAACAACATG  CTTCCTTATA CTCAGATAGT TCACAAACAT TCCATCTTTT ACACGGTGAT GTTGGTGAAG  GTGATGTACC TAGCTACCAG TGTTTCTATT TTTTACGCCA CTCACCGGAT ATTCGACCAA  GGAAACTTTG CACTCTACGG ATACGATGTT CTAATGAGCA TACCACAGGA AACAAGCTAT  AAAGTGATGG ACACAATCTT CCCTAAAATG GTTGGCTGTG AGATCAACAT GTGGGGCCGG  ACTGGCGAAC AGAGCGAATC TCTTCTGTGT GTCCTCCCTC AAAACATCGG CAACCAATAC  TTCTTCCTTA TATTCTGGTT TCTCCTGATT CTCACCATAC TTTCCAACTG TATCTCTGTA  ATAGTGACCA TATTCAGATT TATATTCGTT AGTGGGAGCT ACAAAAGGTT CCTGGCTACC  AGCCTCTTGA ATCACGAAGA ACGATACAAG CTGGTGTTTA CACATGTCGG CACGACTGGA  AGATACATTT TACTGCTCTG TGCCGATCAT AGCAACCCCA AAATATTCGA GGATCTTCTA  GAGATCGTCT GTTCCCTTCT CATAGCAAAC TATCACAAAA GAAAGAGGAG TCGGGATAAG  GGACACAGTC GAGCGGAGGG GGTAGGGACT AAAGGGCGAC ACGGTCTGTC TTTTGTGGAC  TCAACCGTGT GA |
| INXD  (GC: 46.80%) | ATGCTGATCT CGAGCTTAGT TCAGTTCAGC AGGTTATCTC CTTTTAAGGA GATAACTATA  GATGACGGGT GGGACCAACT TAACAGGAGT TTCATGTTCG TTCTGATGGT TATCTGTGGA  ACTATCGTCA CTGTCCGACA ACATACAGGT AACATCATCT CGTGTAACGG TTTCACAAAA  TACGACGGAT CCTTCTCCGA GGACTACTGC TGGACGCAGG GACTCTACAC GATCAGGGAG  GCGTACCACG TGAGCGACGT CAACGTCCCT TATCCCGGAG TTATCCCGGA GGAGATCCCA  CTCTGTCTAG GAGACAATTG TGATAAGCTA GCAAACAGCA ACACCACTCG AGTGTATCAT  CTGTGGTACC AGTGGATCCC CTTCTACTTC TGGCTCGCTT CCGCCGCCTT CTTCCTCCCT  TATCTGATCT ACAAGAGATA CGGATTTGGA GATATCAAGC CTCTGATCCA CATGCTGTAC  AATCCTCTCG ACGGGGACGA AGGAGTGAAG GCAGATTCGG AGAAGGCCTC AATCTGGCTT  TATCACAGAT TCTCTATCTA CATGAACGAG CATTCCATGT ACGCCAACTT TATGGAGAGA  CACGGAATCG GCATTCTCGT TATCGCTATC AAGGTGATGT ACCTGATCAT CTCCGTCCTA  CTCATGGTCA TGACCGCCAT GATGTTCGAG CTGGCTGACT TCAAGCAGTA CGGTATTGTG  TGGGCCCAAC AGTGGCCTGA CCCTCCTGCC AATGTCACAG GAATCAAGGA CCTGCTCTTC  CCCAAGATGG TTGCTTGCGA GATCAAGAGA TGGGGACCTA CTGGTCTGGA GGACGAGAAC  GGAATGTGTG TCCTGGCCCC CAACGTCATC AACCAGTACA TATTCCTCAT CCTCTGGTGG  GCCCTTGTTT TCACCATTGT CTCTAACGTT TTCAACGTAC TGGCTGGAGT TATAAGAATC  GTCTTCATCT ATGGTTCTTA CCGCCGGATG TTGGCTAGCG CTTTCCTCAG AGATGATCCT  CATTACAAGA AGGTCTACTA CAAGATCGGC ACCTCCGGTC GGGTTATCCT GAACATGCTG  GCAGCCTCCA TCTCTCCGAC CTGCTTCCAG GAGATCATGA ACAACGTCTG TCCGCGTCTC  ATCCGGGCCC ACGTCTCCAA GAAGGGACGA AACCTGGGCG ACGACCCCCT GTTGTAG |

**Supplementary Table 3. Names/accessions of ctenophore innexins**

| **FAMILY** | ***M. leidyi*** | ***B. ovata*** | ***P. bachei*** | ***H. californensis*** |
| --- | --- | --- | --- | --- |
| **INXA** | ML25993a |  |  | Hcv1.av93.c10.g19.i1 |
| **INXB** | ML25997a | Bova4.221718.t1 | Pbac.3479642 | Hcv1.av93.c10.g246.i1 |
| **INXC** | ML25998a | Bova4.221717.t1 | Pbac.3473233 | Hcv1.av93.c10.g247.i1 |
| **INXD** | ML25999a | Bova4.221716.t1 | Pbac.3463885 | Hcv1.av93.c10.g248.i1 |
| **INXE** |  |  | Pbac.3471871 | Hcv1.av93.c8.g510.i1 |
| **INXF** |  |  |  | **F1:** Hcv1.av93.c9.g355.i1 |
|  |  |  |  | **F2:** Hcv1.av93.c9.g357.i1 |
| **INXG** | **G1:**ML32831a | Bova4.100819.t1 |  | Hcv1.av93.c10.g192.i1 |
|  | **G2:**ML47742a |  |  |  |
| **INXH** | ML218922a | Bova4.183840.t1 |  | Hcv1.av93.c12.g446.i1 |
| **INXJ** | ML129317a |  | **J1:**Pbac.3466313 | **J1a:**Hcv1.av93.c6.g103.i1 |
|  |  |  |  | **J1b:**Hcv1.av93.c6.g104.i1 |
|  |  |  | **J2:**Pbac.3464407 | **J2:**Hcv1.av93.c1.g815.i1 |
| **INXK** |  | Bova4.221914.t1 |  | Hcv1.av93.c1.g143.i1 |
| **INXL** | ML07312a | Bova4.303225.t1 | Pbac.3465668 | Hcv1.av93.c3.g73.i1 |
| **INXM** | ML223536a | Bova4.301137.t1 | Pbac.346454 | Hcv1.av93.c1.g681.i4 |
| **INXN** |  | Bova4.205910.t1 | Pbac.3465979* | Hcv1.av93.c10.g18.i1 |
| **INXO** | ML036514a | Bova4.177823.t1 |  | Hcv1.av93.c1.g857.i1 |
| **INXP** | ML078817a | Bova4.007720.t1 |  |  |
| **INXQ** |  | Bova4.11432.t1 |  | Hcv1.av93.c12.g447.i1 |
| **INXR** |  | Bova4.00381.t1 |  | Hcv1.av93.c13.g362.i1 |

**Supplementary Table 4. Counts of innexin expression in metacells from Sebé-Pedrós (2018) single cell RNA-Seq data, sorted by percentage of cells in a metacell expressing the gene (in parentheses).**

| **INX ID** | **ML2.2 ID** | **ClusterID (cell type): # cells expressing (% cells in cluster expressing innexin)** |
| --- | --- | --- |
| INXA | ML25993a | C52 (colloblastA): 37 (52%)  C55 (putative tentacle neuron): 3 (4%)  C31 (putative aboral organ neuron): 2 (3%)  C26 (unidentified): 4 (2%)  C27 (putative aboral organ neuron): 3 (2%)  C24 (epithelial): 2 (2%)  C4 (digestive): 2 (2%)  C17 (epithelial): 2 (2%)  C48 (comb cells): 2 (2%)  C49 (comb cells): 3 (1%)  C41 (unidentified): 1 (1%)  C47 (muscle): 1 (1%)  C43 (muscle): 1 (<1%)  C5 (digestive): 1 (1%)  C32 (putative aboral organ neuron): 1 (1%)  C39 (putative aboral organ neuron): 1 (1%)  C53 (colloblastB): 1 (1%)  C42 (unidentified): 1 (1%)  C15 (epithelial): 1 (1%)  C25 (epithelial): 1 (<1%)  C3 (digestive): 1 (<1%)  C21 (epithelial): 1 (<1%)  unassigned (unidentified): 2 |
| INXB | ML25997a | C17 (epithelial): 69 (88%)  C4 (digestive): 74 (86%)  C18 (epithelial): 60 (86%)  C25 (epithelial): 99 (86%)  C19 (epithelial): 35 (79%)  C27 (putative aboral organ neuron): 94 (77%)  C20 (epithelial): 35 (76%)  C13 (epithelial): 67 (76%)  C3 (digestive): 117 (76%)  C44 (unidentified): 50 (76%)  C16 (epithelial): 39 (73%)  C14 (epithelial): 84 (72%)  C21 (epithelial): 95 (68%)  C7 (digestive): 93 (67%)  C29 (putative aboral organ neuron): 82 (66%)  C23 (epithelial): 69 (66%)  C26 (unidentified): 94 (65%)  C28 (putative aboral organ neuron): 102 (64%)  C15 (epithelial): 38 (61%)  C24 (epithelial): 48 (60%)  C42 (unidentified): 58 (57%)  C6 (digestive): 37 (56%)  C41 (unidentified): 52 (54%)  C10 (digestive): 20 (52%)  C36 (putative aboral organ neuron): 33 (52%)  C39 (putative aboral organ neuron): 24 (47%)  C43 (muscle): 87 (46%)  C35 (putative gut/mouth neuron): 26 (45%)  C47 (muscle): 33 (44%)  C9 (digestive): 36 (43%)  C34 (putative pharyngeal neuron): 43 (41%)  C12 (digestive): 23 (40%)  C2 (digestive): 48 (39%)  C8 (digestive): 33 (39%)  C32 (putative aboral organ neuron): 22 (39%)  C52 (colloblastA): 26 (37%)  C55 (putative tentacle neuron): 25 (35%)  C54 (tentacle): 15 (35%)  C40 (putative aboral organ neuron): 32 (35%)  C38 (unidentified): 27 (35%)  C51 (photocytes): 21 (34%)  C22 (epithelial): 18 (33%)  C37 (unidentified): 16 (32%)  C11 (digestive): 22 (30%)  C5 (digestive): 16 (30%)  C48 (comb cells): 21 (27%)  C1 (digestive): 43 (27%)  C33 (putative nerve net neuron): 35 (25%)  C30 (putative aboral organ neuron): 7 (20%)  C31 (putative aboral organ neuron): 12 (20%)  C53 (colloblastB): 19 (19%)  C45 (muscle): 15 (17%)  C49 (comb cells): 27 (17%)  C50 (comb cells): 11 (13%)  C46 (muscle): 9 (13%)  unassigned: 284 |
| INXC | ML25998a | C30 (putative aboral organ neuron): 3 (8%)  C35 (putative gut/mouth neuron): 4 (7%)  C33 (putative nerve net neuron): 8 (5%)  C34 (putative pharyngeal neuron): 5 (4%)  C16 (epithelial): 2 (3%)  C10 (digestive): 1 (2%)  C49 (comb cells): 2 (1%)  C40 (putative aboral organ neuron): 1 (1%)  C13 (epithelial): 1 (1%)  C25 (epithelial): 2 (1%)  C50 (comb cells): 1 (1%)  C28 (putative aboral organ neuron): 2 (1%)  C27 (putative aboral organ neuron): 2 (1%)  C48 (comb cells): 1 (1%)  C7 (digestive): 1 (<1%)  C14 (epithelial): 1 (<1%)  C26 (unidentified): 1 (<1%)  unassigned: 5 |
| INXD | ML25999a | C25 (epithelial): 105 (92%)  C44 (unidentified): 57 (87%)  C13 (epithelial): 72 (81%)  C4 (digestive): 69 (80%)  C17 (epithelial): 63 (80%)  C27 (putative aboral organ neuron): 98 (80%)  C14 (epithelial): 91 (78%)  C18 (epithelial): 53 (76%)  C26 (unidentified): 107 (74%)  C20 (epithelial): 34 (73%)  C3 (digestive): 107 (70%)  C21 (epithelial): 94 (67%)  C7 (digestive): 92 (66%)  C29 (putative aboral organ neuron): 78 (62%)  C28 (putative aboral organ neuron): 95 (60%)  C15 (epithelial): 37 (59%)  C24 (epithelial): 44 (55%)  C19 (epithelial): 24 (54%)  C6 (digestive): 35 (53%)  C16 (epithelial): 28 (52%)  C23 (epithelial): 54 (52%)  C36 (putative aboral organ neuron): 32 (50%)  C42 (unidentified): 49 (49%)  C9 (digestive): 37 (45%)  C12 (digestive): 24 (42%)  C2 (digestive): 52 (42%)  C43 (muscle): 78 (41%)  C47 (muscle): 29 (39%)  C8 (digestive): 32 (38%)  C52 (colloblastA): 26 (37%)  C11 (digestive): 26 (36%)  C54 (tentacle): 15 (35%)  C41 (unidentified): 34 (35%)  C22 (epithelial): 19 (35%)  C5 (digestive): 18 (34%)  C39 (putative aboral organ neuron): 17 (33%)  C32 (putative aboral organ neuron): 18 (32%)  C48 (comb cells): 25 (32%)  C40 (putative aboral organ neuron): 28 (31%)  C10 (digestive): 12 (31%)  C55 (putative tentacle neuron): 22 (30%)  C51 (photocytes): 18 (29%)  C30 (putative aboral organ neuron): 10 (28%)  C1 (digestive): 43 (27%)  C38 (unidentified): 21 (27%)  C50 (comb cells): 22 (26%)  C37 (unidentified): 12 (24%)  C35 (putative gut/mouth neuron): 14 (24%)  C34 (putative pharyngeal neuron): 22 (21%)  C45 (muscle): 18 (21%)  C49 (comb cells): 27 (17%)  C33 (putative nerve net neuron): 21 (15%)  C46 (muscle): 7 (10%)  C31 (putative aboral organ neuron): 6 (10%)  C53 (colloblastB): 9 (9%)  unassigned: 241 |
| INXG.1 | ML32831a | C34 (putative pharyngeal neuron): 57 (54%)  C50 (comb cells): 26 (31%)  C48 (comb cells): 19 (24%)  C47 (muscle): 14 (18%)  C33 (putative nerve net neuron): 24 (17%)  C49 (comb cells): 28 (17%)  C18 (epithelial): 8 (11%)  C32 (putative aboral organ neuron): 4 (7%)  C35 (putative gut/mouth neuron): 3 (5%)  C27 (putative aboral organ neuron): 7 (5%)  C30 (putative aboral organ neuron): 2 (5%)  C20 (epithelial): 2 (4%)  C21 (epithelial): 6 (4%)  C40 (putative aboral organ neuron): 4 (4%)  C2 (digestive): 4 (3%)  C39 (putative aboral organ neuron): 2 (3%)  C55 (putative tentacle neuron): 2 (2%)  C29 (putative aboral organ neuron): 3 (2%)  C17 (epithelial): 2 (2%)  C28 (putative aboral organ neuron): 4 (2%)  C46 (muscle): 2 (2%)  C25 (epithelial): 3 (2%)  C51 (photocytes): 1 (1%)  C44 (unidentified): 1 (1%)  C1 (digestive): 2 (1%)  C24 (epithelial): 1 (1%)  C45 (muscle): 1 (1%)  C13 (epithelial): 1 (1%)  C41 (unidentified): 1 (1%)  C4 (digestive): 1 (1%)  C7 (digestive): 1 (<1%)  C43 (muscle): 1 (<1%)  C23 (epithelial): 1 (<1%)  C14 (epithelial): 1 (<1%)  C3 (digestive): 1 (<1%)  unassigned: 32 |
| INXG.2 | ML47742a | C34 (putative pharyngeal neuron): 14 (13%)  C47 (muscle): 4 (5%)  C33 (putative nerve net neuron): 8 (5%)  C49 (comb cells): 5 (3%)  C13 (epithelial): 1 (1%)  C50 (comb cells): 1 (1%)  C4 (digestive): 1 (1%)  C55 (putative tentacle neuron): 1 (1%)  C9 (digestive): 1 (1%)  C12 (digestive): 1 (1%)  C35 (putative gut/mouth neuron): 1 (1%)  C24 (epithelial): 1 (1%)  C39 (putative aboral organ neuron): 1 (1%)  C17 (epithelial): 1 (1%)  C28 (putative aboral organ neuron): 1 (<1%)  C7 (digestive): 1 (<1%)  C25 (epithelial): 1 (<1%)  unassigned: 6 |
| INXH | ML218922a | C48 (comb cells): 55 (71%)  C49 (comb cells): 90 (56%)  C50 (comb cells): 33 (39%)  C32 (putative aboral organ neuron): 4 (7%)  C24 (epithelial): 5 (6%)  C13 (epithelial): 5 (5%)  C42 (unidentified): 4 (4%)  C27 (putative aboral organ neuron): 5 (4%)  C25 (epithelial): 4 (3%)  C1 (digestive): 5 (3%)  C4 (digestive): 3 (3%)  C36 (putative aboral organ neuron): 2 (3%)  C7 (digestive): 5 (3%)  C31 (putative aboral organ neuron): 2 (3%)  C40 (putative aboral organ neuron): 2 (2%)  C55 (putative tentacle neuron): 2 (2%)  C26 (unidentified): 3 (2%)  C19 (epithelial): 1 (2%)  C53 (colloblastB): 2 (2%)  C21 (epithelial): 4 (2%)  C41 (unidentified): 2 (2%)  C6 (digestive): 1 (1%)  C18 (epithelial): 1 (1%)  C35 (putative gut/mouth neuron): 1 (1%)  C16 (epithelial): 1 (1%)  C45 (muscle): 1 (1%)  C47 (muscle): 1 (1%)  C28 (putative aboral organ neuron): 3 (1%)  C39 (putative aboral organ neuron): 1 (1%)  C2 (digestive): 2 (1%)  C38 (unidentified): 1 (1%)  C43 (muscle): 3 (1%)  C33 (putative nerve net neuron): 2 (1%)  C44 (unidentified): 1 (1%)  C5 (digestive): 1 (1%)  C29 (putative aboral organ neuron): 1 (<1%)  C34 (putative pharyngeal neuron): 1 (<1%)  C3 (digestive): 1 (<1%)  C23 (epithelial): 1 (<1%)  C14 (epithelial): 1 (<1%)  unassigned: 38 |
| INXJ | ML129317a | C27 (putative aboral organ neuron): 16 (13%)  C26 (unidentified): 8 (5%)  C30 (putative aboral organ neuron): 2 (5%)  C28 (putative aboral organ neuron): 6 (3%)  C53 (colloblastB): 3 (3%)  C37 (unidentified): 1 (2%)  C32 (putative aboral organ neuron): 1 (1%)  C17 (epithelial): 1 (1%)  C36 (putative aboral organ neuron): 1 (1%)  C42 (unidentified): 1 (1%)  C14 (epithelial): 1 (<1%)  C2 (digestive): 1 (<1%)  C25 (epithelial): 1 (<1%)  unassigned: 8 |
| INXL | ML07312a | C48 (comb cells): 39 (50%)  C49 (comb cells): 46 (29%)  C50 (comb cells): 22 (26%)  C42 (unidentified): 1 (1%)  C32 (putative aboral organ neuron): 1 (1%)  C41 (unidentified): 1 (1%)  C43 (muscle): 2 (1%)  C52 (colloblastA): 1 (1%)  C4 (digestive): 1 (1%)  C18 (epithelial): 1 (1%)  C28 (putative aboral organ neuron): 3 (1%)  C7 (digestive): 1 (<1%)  C2 (digestive): 1 (<1%)  unassigned: 21 |
| INXM | ML223536a | C48 (comb cells): 17 (22%)  C49 (comb cells): 36 (22%)  C50 (comb cells): 15 (18%)  C35 (putative gut/mouth neuron): 2 (3%)  C30 (putative aboral organ neuron): 1 (2%)  C37 (unidentified): 1 (2%)  C31 (putative aboral organ neuron): 1 (1%)  C15 (epithelial): 1 (1%)  C46 (muscle): 1 (1%)  C11 (digestive): 1 (1%)  C43 (muscle): 3 (1%)  C25 (epithelial): 2 (1%)  C6 (digestive): 1 (1%)  C51 (photocytes): 1 (1%)  C36 (putative aboral organ neuron): 1 (1%)  C27 (putative aboral organ neuron): 1 (<1%)  unassigned: 10 |
| INXO | ML036514a | C34 (putative pharyngeal neuron): 33 (31%)  C33 (putative nerve net neuron): 32 (23%)  C47 (muscle): 17 (22%)  C30 (putative aboral organ neuron): 5 (14%)  C35 (putative gut/mouth neuron): 6 (10%)  C46 (muscle): 6 (8%)  C17 (epithelial): 7 (8%)  C55 (putative tentacle neuron): 5 (7%)  C45 (muscle): 6 (7%)  C51 (photocytes): 4 (6%)  C18 (epithelial): 4 (5%)  C25 (epithelial): 5 (4%)  C20 (epithelial): 2 (4%)  C42 (unidentified): 3 (3%)  C48 (comb cells): 3 (3%)  C6 (digestive): 2 (3%)  C32 (putative aboral organ neuron): 2 (3%)  C19 (epithelial): 1 (2%)  C4 (digestive): 2 (2%)  C24 (epithelial): 2 (2%)  C40 (putative aboral organ neuron): 2 (2%)  C29 (putative aboral organ neuron): 3 (2%)  C13 (epithelial): 2 (2%)  C37 (unidentified): 1 (2%)  C38 (unidentified): 1 (1%)  C53 (colloblastB): 1 (1%)  C36 (putative aboral organ neuron): 1 (1%)  C41 (unidentified): 1 (1%)  C44 (unidentified): 1 (1%)  C31 (putative aboral organ neuron): 1 (1%)  C14 (epithelial): 2 (1%)  C50 (comb cells): 1 (1%)  C27 (putative aboral organ neuron): 2 (1%)  C2 (digestive): 2 (1%)  C49 (comb cells): 2 (1%)  C21 (epithelial): 1 (<1%)  C7 (digestive): 1 (<1%)  C43 (muscle): 1 (<1%)  unassigned: 14 |
| INXP | ML078817a | C30 (putative aboral organ neuron): 2 (5%)  C34 (putative pharyngeal neuron): 4 (3%)  C39 (putative aboral organ neuron): 2 (3%)  C20 (epithelial): 1 (2%)  C13 (epithelial): 1 (1%)  C35 (putative gut/mouth neuron): 1 (1%)  C33 (putative nerve net neuron): 2 (1%)  C41 (unidentified): 1 (1%)  C36 (putative aboral organ neuron): 1 (1%)  C25 (epithelial): 1 (<1%)  C28 (putative aboral organ neuron): 1 (<1%)  unassigned: 5 |

**Supplementary Table 5. Counts of occurrences of cells with N innexins in Sebé-Pedrós (2018) single cell RNA-Seq data**

| **Number of occurrences of innexins (N)** | **Count of cells with N innexins (total: 6144)** | **Percentage of cells with N innexins** |
| --- | --- | --- |
| 0 | 2301 | 37.5% |
| 1 | 1724 | 28.1% |
| 2 | 1749 | 28.5% |
| 3 | 271 | 11.7% |
| 4 | 67 | 1.1% |
| 5 | 23 | 0.4% |
| 6 | 5 | 0.1% |
| 7 | 3 | < 0.1% |
| 8 | 0 | 0% |
| 9 | 1 | < 0.1% |

**Supplementary Table 6. Counts of innexin co-expression from Sebé-Pedrós (2018) single cell RNA-Seq data**

|  | **INXB(2720)** | **INXJ(51)** | **INXP(22)** | **INXG.2(51)** | **INXG.1(272)** | **INXD(2521)** | **INHX(301)** | **INXC(43)** | **INXM(95)** | **INXL(141)** |
| --- | --- | --- | --- | --- | --- | --- | --- | --- | --- | --- |
| **INXA(74)** | 28(0.378) | 0(0.000) | 0(0.000) | 0(0.000) | 1(0.014) | 28(0.378) | 2(0.027) | 0(0.000) | 1(0.014) | 1(0.014) |
| **INXL(141)** | 46(0.326) | 1(0.020) | 2(0.091) | 6(0.118) | 44(0.312) | 52(0.369) | 100(0.709) | 3(0.070) | 37(0.389) |  |
| **INXM(95)** | 37(0.389) | 0(0.000) | 0(0.000) | 3(0.059) | 27(0.284) | 29(0.305) | 60(0.632) | 3(0.070) |  |  |
| **INXC(43)** | 23(0.535) | 1(0.023) | 2(0.091) | 5(0.116) | 10(0.233) | 23(0.535) | 5(0.116) |  |  |  |
| **INXH(301)** | 110(0.365) | 1(0.020) | 2(0.091) | 9(0.176) | 72(0.265) | 113(0.375) |  |  |  |  |
| **INXD(2521)** | 1761(0.699) | 43(0.843) | 10(0.455) | 20(0.392) | 117(0.430) |  |  |  |  |  |
| **INXG.1(272)** | 123(0.452) | 1(0.020) | 6(0.273) | 32(0.627) |  |  |  |  |  |  |
| **INXG.2(51)** | 31(0.608) | 0(0.000) | 4(0.182) |  |  |  |  |  |  |  |
| **INXP(22)** | 14(0.636) | 0(0.000) |  |  |  |  |  |  |  |  |
| **INXJ(51)** | 40(0.784) |  |  |  |  |  |  |  |  |  |

**Supplemental Video 1. Contractile activity of the *Mnemiopsis Leidyi* muscle cell.** Activity of the cell was recorded simultaneously with electrophysiological recording (Supplemental Figure 3). Glutamate repetitively evokes the cell contractions. Video - 50 fps. Actual sampling frequency – 4Hz.

**References**

DeBiasse, Melissa B., and Joseph F. Ryan. "Phylotocol: promoting transparency and overcoming bias in phylogenetics." Systematic biology 68.4 (2019): 672-678.

Tazuke SI, Schulz C, Gilboa L, Fogarty M, Mahowald AP, Guichet A, Ephrussi A, Wood CG, Lehmann R, Fuller MT. A germline-specific gap junction protein required for survival of differentiating early germ cells. Development. 2002 May;129(10):2529-39. doi: 10.1242/dev.129.10.2529.
